# Polymorphism at rs9264942 is associated with HLA-C expression and inflammatory bowel disease in the Japanese

**DOI:** 10.1038/s41598-020-69370-8

**Published:** 2020-07-24

**Authors:** Hiroshi Suzuki, Satoru Joshita, Atsuhiro Hirayama, Akihiro Shinji, Kenji Mukawa, Minako Sako, Naoki Yoshimura, Tomoaki Suga, Takeji Umemura, Norihiro Ashihara, Tomoo Yamazaki, Masao Ota

**Affiliations:** 1 Department of Medicine, Division of Gastroenterology and Hepatology, Shinshu University School of Medicine, 3-1-1 Asahi, Matsumoto, Nagano 390-8621 Japan; 2 Department of Gastroenterology, Japanese Red Cross Society Suwa Red Cross Hospital, Suwa, Japan; 3 Department of Medical Oncology, Japanese Red Cross Society Suwa Red Cross Hospital, Suwa, Japan; 4 Center for Inflammatory Bowel Disease, Tokyo Yamate Medical Center, Tokyo, Japan; 5 Department of Inflammatory Bowel Disease, Yokohama City University Medical Center, Yokohama, Japan; 6 Department of Life Innovation, Institute for Biomedical Sciences, Shinshu University, Matsumoto, Japan

**Keywords:** Genetics, Immunology, Gastroenterology

## Abstract

An expression quantitative trait locus (eQTL) single-nucleotide polymorphism (SNP) at rs9264942 was earlier associated with human leukocyte antigen (HLA)-C expression in Europeans. HLA-C has also been related to inflammatory bowel disease (IBD) risk in the Japanese. This study examined whether an eQTL SNP at rs9264942 could regulate HLA-C expression and whether four SNP haplotypes, including the eQTL SNP at rs9264942 and three SNPs at rs2270191, rs3132550, and rs6915986 of IBD risk carried in the HLA-C*12:02~B*52:01~DRB1*15:02 allele, were associated with IBD in the Japanese. HLA-C expression on CD3e^+^CD8a^+^ lymphocytes was significantly higher for the CC or CT genotype than for the TT genotype of rs9264942. The TACC haplotype of the four SNPs was associated with a strong susceptibility to ulcerative colitis (UC) but protection against Crohn’s disease (CD) as well as with disease clinical outcome. While UC protectivity was significant but CD susceptibility was not for the CGTT haplotype, the significance of UC protectivity disappeared but CD susceptibility reached significance for the CGCT haplotype. In conclusion, our findings support that the eQTL SNP at rs9264942 regulates HLA-C expression in the Japanese and suggest that the four SNPs, which are in strong linkage disequilibrium, may be surrogate marker candidates of a particular HLA haplotype, HLA-C*12:02~B*52:01~DRB1*15:02, related to IBD susceptibility and disease outcome.

## Introduction

Inflammatory bowel disease (IBD) is a chronic or remission-relapse inflammatory disease of the gastrointestinal tract. IBD includes two types of chronic gut disorders: ulcerative colitis (UC) and Crohn’s disease (CD)^1^. UC primarily affects the mucous membranes and often forms erosions and ulcers in the colon, which can lead to inflammation of the rectum and extend proximally. There are three UC phenotypes in the clinical setting: proctitis, left-sided colitis, and pancolitis^2^. CD produces granulomatous inflammation with ulceration and fibrosis mainly in the small intestine, but also anywhere in the digestive tract. Long-term severe intestinal inflammation can result in ulcers, constriction, and perforation of the intestinal tract. Indeed, IBD chronically injures the digestive tract, leading to tissue damage, function loss, disability, and systemic inflammation^1^.

The exact etiology of IBD remains unknown, although a multifactorial pathogenesis that includes genetic, immunological, environmental, and microbial factors are likely involved^3–7^. Specifically, the influence of genetic factors on IBD pathogenesis has been supported by the high concordance rate among monozygotic twins as well as the high relative risk in affected siblings^8,9^.

To date, genome-wide association studies and the candidate-gene approach have identified more than 240 IBD susceptibility genes or loci outside of the human leukocyte antigen (HLA) region, including *nucleotide oligomerization domain 2*, *autophagy related 16-like 1*, *interleukin-23*, *PR domain-containing 1*, and *caspase recruitment domain 9*^3,10–17^. Among the many candidate genes proposed, however, the HLA region has been consistently and strongly associated with IBD onset. HLA class I proteins play a crucial role in human immune responses^18–20^. High expression levels of HLA-C molecules, which contribute to viral control in HIV-infected individuals, reportedly have a deleterious effect on CD in patients of European descent^21^. Moreover, among several single-nucleotide polymorphisms (SNPs) associated with HLA-C expression^22,23^, a SNP located 35 kb upstream of the coding region of the *HLA-C* gene in chromosome 6 (-35C/T; rs9264942) was suggested to play an important role in controlling HLA-C cell surface molecule expression as an expression quantitative trait locus (eQTL) in subjects of European ancestry^24^. However, no such association studies on Japanese IBD patients have been published^24^.

Including HLA-DQB1, HLA-DRB1, and HLA-DQA1, the HLA class II region has also been associated with IBD onset across human groups^7,25,26^. A particular haplotype, HLA-C*12:02~B*52:01~DRB1*15:02, was related to an increased risk for UC but a reduced risk for CD in a Japanese population^25^. Specifically, both HLA-B*52:01 and HLA-DRB1*15:02 alleles were directly associated with UC susceptibility and CD protection in the Japanese, whereas HLA-C genetic involvement in IBD remained unknown^25^. Since HLA genes are the most highly polymorphic in the human genome, a high-resolution HLA and SNP haplotype map analysis was developed for disease association studies^27–29^. Based on that dataset and Okada’s report^25^, three SNPs at rs2270191, rs3132550, and rs6915986 have strong linkage disequilibrium (LD) with the HLA-C*12:02 (r^2^ = 1), HLA-B*52:01 (r^2^ = 0.94), and HLA-DRB1*15:02 (r^2^ = 0.89) alleles, respectively, in Japanese populations^22,25^ (Supplementary Fig. 1). However, no association studies of these tag SNPs have been reported in terms of Japanese IBD.

The aim of this study was to determine whether the eQTL SNP at rs9264942 could regulate HLA-C expression in the Japanese and analyze the relationships of SNPs in strong LD of a particular HLA haplotype with UC and CD susceptibility. It sought to uncover important findings on IBD from the in vivo, genetic, and functional aspects in well-defined patient groups and controls.

## Results

### Comparisons of HLA-C expression on peripheral blood mononuclear cells by the eQTL rs9264942 SNP genotype in healthy Japanese subjects

**Figure 1:**
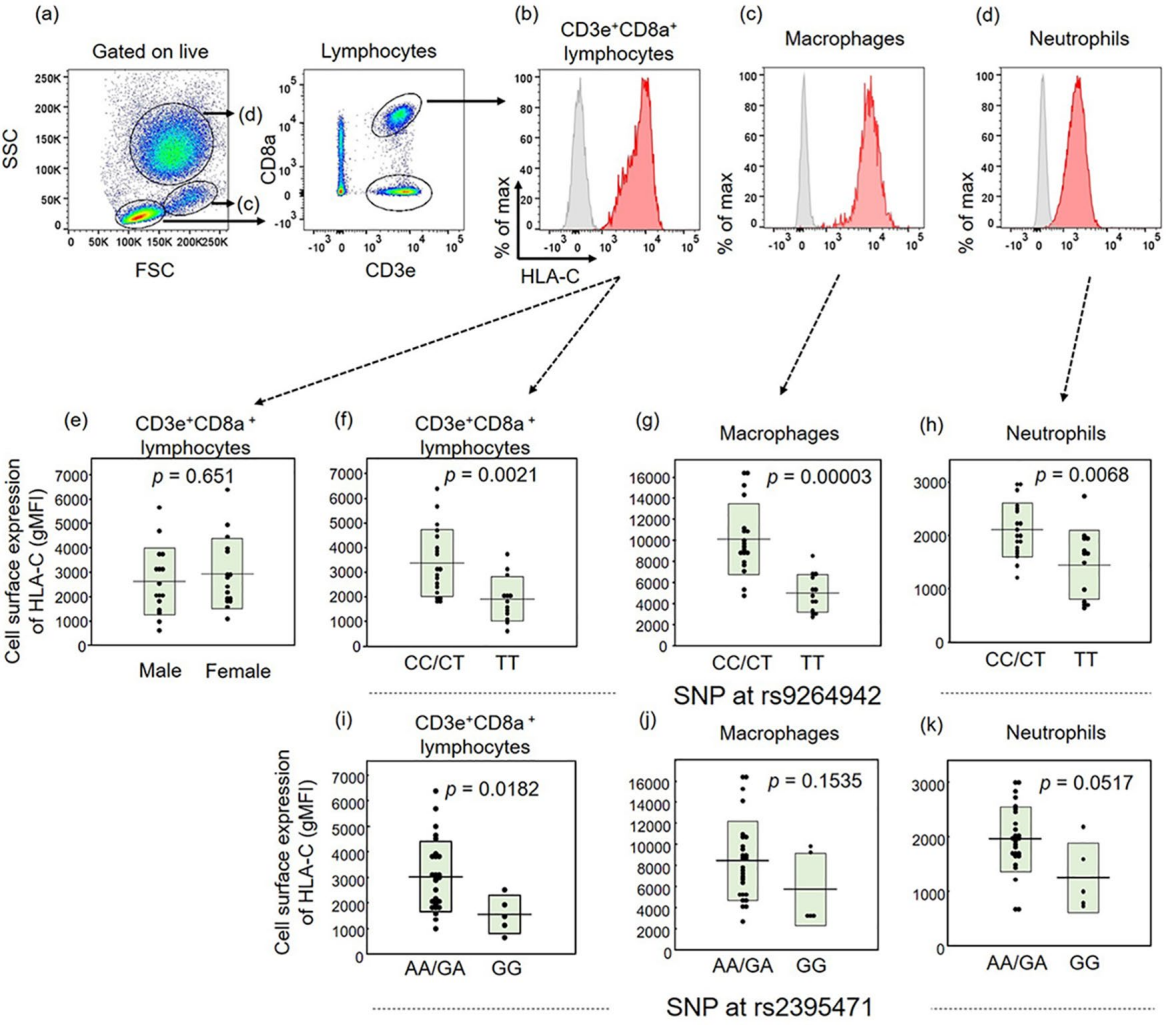
HLA-C expression on PBMC by flow cytometry analysis. Representative gating strategy (**a**). Representative HLA-C expression on CD3e^+^CD8a^+^ lymphocytes (**b**), macrophages (**c**), and neutrophils (**d**) (red) in relation to the isotype control (gray). Comparisons of geometric mean fluorescence intensity quantification of the cell surface expression of HLA-C on CD3e^+^CD8a^+^ lymphocytes between males and females (**e**) and between the SNP of the CC or CT genotype and of the TT genotype at rs9264942 on CD3e^+^CD8a^+^ lymphocytes (**f**), macrophages (**g**), and neutrophils (**h**) as well as between the SNP of the AA or AG genotype and the GG genotype at rs2395471 on CD3e^+^CD8a^+^ T lymphocytes (**i**), macrophages (**j**), and neutrophils (**k**).

A total of 32 healthy control subjects were included for the analysis of HLA-C expression on peripheral blood mononuclear cells (PBMC) (Table 1). The gating strategy for PBMC is shown in Fig. 1a. Although the cell surface expression of HLA-C on CD3e^+^CD8a^+^ T lymphocytes (Fig. 1b) as detected by flow cytometry in healthy subjects was comparable between males and females (Fig. 1e), it was significantly higher for the CC or CT genotype than for the TT genotype at rs9264942. HLA-C expression on CD3e^+^CD8a^+^ T lymphocytes (Fig. 1f), macrophages (Fig. 1c,g), and neutrophils (Fig. 1d,h) were significantly higher for the CC or CT genotype than for the TT genotype. Since another SNP at rs2395471 was also reported to be an eQTL SNP of HLA-C^23^, we compared HLA-C expression for the AA or AG genotype and the GG genotype at rs2395471 on PBMC. HLA-C expression on CD3e^+^CD8a^+^ T lymphocytes (Fig. 1i) was significantly higher for the AA or AG genotype than for the GG genotype at rs2395471, while HLA-C expression on macrophages (Fig. 1j) and neutrophils (Fig. 1k) were comparable between the groups.

**Table 1:** Age and gender proportions of healthy subjects for HLA-C expression analysis.

Characteristic		Genotype of SNP at rs9264942			
	All	CC	CT	TT	CC or CT vs. TT
	(n = 32)	(n = 7)	(n = 12)	(n = 13)	*p *value
Age, median years (first-third quartile)	35 (26–67)	40 (27–58)	40 (29–67)	31 (26–45)	0.094
Male / female	16/16	2/5	4/8	10/3	0.012

*HLA* human leukocyte antigen, *SNP* single-nucleotide polymorphism.

### Association of the eQTL rs9264942 SNP of HLA-C expression with IBD susceptibility

A total of 160 patients with UC and 275 patients with CD along with 325 healthy subjects were enrolled for this association study (Table 2). The C allele frequency of the eQTL SNP at rs9264942, which has been associated with HLA-C expression, was significantly higher in UC patients than in the healthy control group (UC vs. controls: 49.7% vs. 35.5%, odds ratio [OR] 1.79; *pc* = 9.52 × 10^–4^) (Table 3). In terms of clinical findings, there were no significant differences for UC between the CC or CT genotype and the TT genotype (Supplementary Table 1). There was also no remarkable difference for the C allele frequency of the rs9264942 SNP between the CD and healthy control groups (OR 1.00; *pc* = 1.000) (Table 3).

**Table 2:** Demographic and clinical data of UC, CD, PBC and healthy subjects.

Characteristic	UC	CD	PBC	Control	UC versus control	CD versus control	PBC versus control
	(n = 160)	(n = 275)	(n = 328)	(n = 325)	*p* value	*p* value	*p* value
Age, years	48 (19–84)	41 (13–87)	71 (18–95)	47 (29–87)	0.880	0.157	3.83 × 10^–72^
Age of onset, median years (first-third quartile)	34 (9–76)	23 (7–64)					
Male/female	83/77	190/85	53/275	90/235	1.72 × 10^–7^	4.16 × 10^–24^	3.70 × 10^–4^
Disease phenotype							
Location*	31/55/73/1	45/48/182/0					
PSL administration	84 (53.2%)	108 (39.3%)					
Anti-TNF-α administration	47 (29.7%)	150 (54.5%)					
Surgical history	12 (7.5%)	176 (64%)					
Presence of anal lesion	-	177 (64.4%)					
Family history of IBD	5 (3.1%)	26 (9.5%)					
Liver cirrhosis			48 (14.6%)				
ANA (× 40 <)			209 (63.7%)				
AMA			265 (80.8%)				

*Defined as colonic/ileal/ileocolonic/not determined in CD and proctitis/left-sided colitis/pancolitis/not determined in UC.

*UC* ulcerative colitis, *CD* Crohn’s disease, *PBC* primary biliary cholangitis, *PSL* prednisolone, *ANA* anti-nuclear antibody, *AMA* anti-mitochondrial antibody.

**Table 3:** Association of four SNPs with IBD susceptibility.

SNP	Position	Allele	UC (n = 160)	CD (n = 275)	Control (n = 325)	PBC (n = 328)	UC versus control	CD versus control	PBC versus control
			Minor allele frequencies				*pc*-value OR (95% CI)	*pc*-value OR (95% CI)	*pc-*value OR (95% CI)
rs2270191	31112543	T<C	0.256	0.067	0.117	0.111	1.16 × 10^–7^ 2.61 (1.85–3.69)	0.014 0.53 (0.36–0.82)	1.000 0.95 (0.68–1.34)
rs3132550	31118271	A<G	0.269	0.069	0.120	0.117	2.17 × 10^–8^ 2.70 (1.92–3.81)	0.012 0.55 (0.36–0.82)	1.000 0.98 (0.7–1.37)
rs9264942	31306603	C<T	0.497	0.355	0.355	0.352	9.52 × 10^–4^ 1.79 (1.37–2.35)	1.000 1.00 (0.79–1.26)	1.000 0.99 (0.79–1.24)
rs6915986	32716121	C<T	0.231	0.058	0.112	0.099	4.28 × 10^–6^ 2.61 (1.85–3.69)	0.004 0.49 (0.32–0.75)	1.000 0.87 (0.61–1.24)

*SNP* single-nucleotide polymorphism, *IBD* inflammatory bowel disease, *UC* ulcerative colitis, *CD* Crohn’s disease, *PBC* primary biliary cholangitis, *pc* corrected *p* value (Bonferroni correction), *OR* odds ratio, *CI* confidence interval.

*p* values were calculated by chi-squared test 2 × 2 contingency tables.

### Association of three SNPs at rs2270191, rs3132550, and rs6915986 with IBD susceptibility

The allele frequencies of three SNPs at rs2270191, rs3132550, and rs6915986 in patients with UC and CD and in healthy and primary biliary cholangitis (PBC) disease controls are shown in Table 3. The frequency of the T allele at rs2270191 in strong LD with the HLA-C*12:02 allele (r^2^ = 1), the A allele at rs3132550 in strong LD with the HLA-B*52:01 allele (r^2^ = 0.94), and the C allele at rs6915986 in strong LD with the HLA-DRB1*15:02 allele (r^2^ = 0.89) were significantly higher in UC patients than in the healthy group but significantly lower in CD patients than in healthy controls (Table 3). These allele frequencies were comparable between healthy and PBC disease controls (Table 3).

### Haplotype analysis of the SNPs at rs2270191, rs3132550, and rs6915986

**Figure 2:**
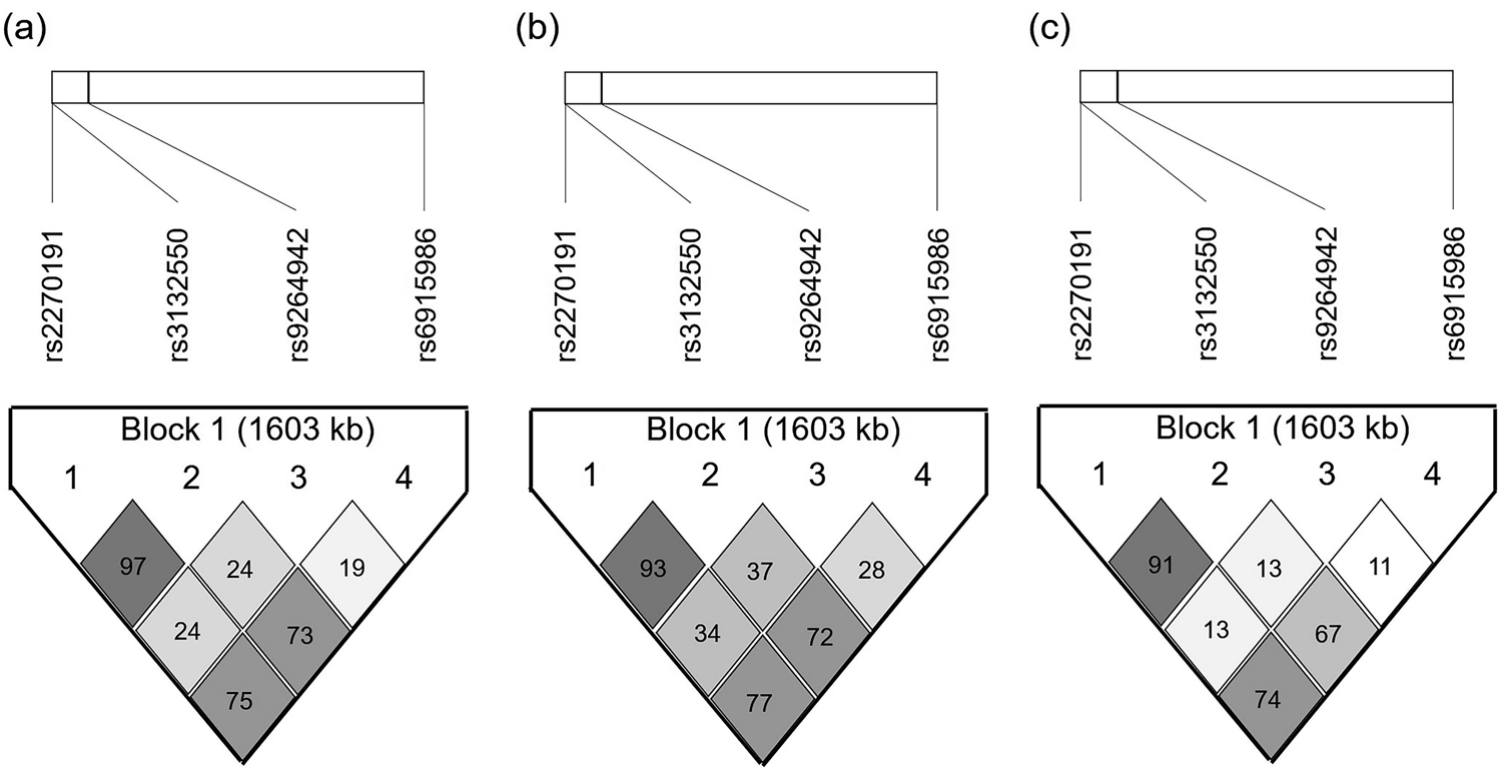
LD plots of four SNPs at rs2270171, rs3132550, rs9264942, and rs6915986 of healthy controls (**a**), ulcerative colitis patients (**b**) and Crohn’s disease patients (**c**). Values of r^2^ corresponding to each pair are expressed as a percentage and shown within the respective square.

Haplotype frequencies were calculated using an expectation–maximization algorithm for the three SNPs (rs2270191, rs3132550, and rs6915986) and compared among UC, CD, PBC, and healthy control groups. The three-SNP haplotype of TAC, which was predicted as associated with the particular HLA haplotype of HLA-C*12:02~B*52:01~DRB1*15:02, showed a strong susceptibility correlation between UC and controls (OR 2.53; *pc* = 3.92 × 10^–7^) but a protective association between CD and controls (OR 0.50; *pc* = 0.002) (Table 4). In contrast, the three-SNP haplotype of CGT showed a strong protectivity correlation between UC and controls (OR 0.39; *pc* = 1.58 × 10^–8^) but a significant susceptibility association between CD and controls (OR 1.90; *pc* = 1.19 × 10^–3^) (Table 4). The three-SNP haplotypes of CGT and TAC accounted for the vast majority of the cohort, which indicated that the SNPs were in strong LD with each other, as shown in Fig. 2. Moreover, these three-SNP haplotype frequencies were comparable between healthy and PBC disease controls (Table 4).

**Table 4:** Association of the three-SNP* haplotype or four-SNP* haplotype with IBD susceptibility.

Haplotype	UC (n = 160)	CD (n = 275)	Control (n = 325)	PBC (n = 328)	UC versus control	CD versus control	PBC versus control
	Haplotype frequencies				*pc*-value OR (95% CI)	*pc*-value OR (95% CI)	*pc-*value OR (95% CI)
3 SNPs*							
CGT	0.721	0.927	0.869	0.873	1.58 × 10^–8^ 0.39 (0.28–0.54)	1.19 × 10^–3^ 1.90 (1.28–2.82)	0.835 1.04 (0.75–1.43)
TAC	0.221	0.053	0.101	0.090	3.92 × 10^–7^ 2.53 (1.76–3.65)	0.002 0.50 (0.32–0.78)	0.487 0.88 (0.61–1.27)
TAT	0.035	0.013	0.015	0.022	0.055 2.29 (0.96–5.44)	0.708 0.83 (0.31–2.20)	0.421 1.40 (0.62–3.18)
CGC	0.010	0.004	0.011	0.009	0.843 0.87 (0.22–3.40)	0.156 0.34 (0.07–1.63)	0.773 0.85 (0.28–2.55)
CAT	0.012	0.004	0.003	0.006	0.078 4.11 (0.75–22.6)	0.862 1.19 (0.17–8.48)	0.417 1.99 (0.36–10.9)
4 SNPs**							
CGTT	0.498	0.645	0.639	0.645	2.66 × 10^–5^ 0.56 (0.42–0.73)	0.828 1.03 (0.81–1.30)	0.828 1.03 (0.82–1.29)
CGCT	0.224	0.284	0.231	0.228	0.803 0.96 (0.70–1.32)	0.042 1.31 (1.01–1.70)	0.933 0.98 (0.76–1.27)
TACC	0.221	0.053	0.101	0.090	3.96 × 10^–7^ 2.53 (1.77–3.62)	0.002 0.50 (0.32–0.78)	0.486 0.88 (0.60–1.27)
TACT	0.035	0.013	0.015	0.022	0.053 2.28 (0.98–5.30)	0.705 0.82 (0.31–2.17)	0.418 1.40 (0.62–3.18)

Haplotypes were created by an accelerated EM algorithm in Haploview software.

*SNP* single-nucleotide polymorphism, *IBD* inflammatory bowel disease, *UC* ulcerative colitis, *CD* Crohn’s disease, *PBC* primary biliary cholangitis, *OR* odds ratio, *CI* confidence interval.

*SNPs at rs2270191, rs3132550, and rs6915986.

**SNPs at rs2270191, rs3132550, rs9264942, and rs6915986.

### Logistic regression analysis of SNPs associated with IBD susceptibility

The strong LD of the three SNPs at rs2270191, rs3132550, and rs6915986 indicated that simultaneous logistic regression analysis to determine which SNP was independently associated with IBD susceptibility was inappropriate due to multi-collinearity. However, since rs9264942 SNP distribution was different from that of the other three SNPs regardless of being within a haplotype block (Fig. 2 and Supplementary Fig. 1), the SNP at rs9264942 was compared with the representative SNP at rs3132550 by logistic regression analysis. The SNP at rs3132550 was independently associated with UC susceptibility [OR 3.507, 95% confidence interval (95% CI)] 2.176–5.654; *p* < 0.00001), whereas the SNP at rs9264942 did not reach statistical significance (OR 1.236, 95% CI 0.744–2.054; *p* = 0.413). Moreover, the SNP at rs3132550 was independently associated with CD protectivity (OR 0.478, 95% CI 0.298–0.767; *p* = 0.00221), unlike the SNP at rs9264942 (OR 1.376, 95% CI 0.966–1.961; *p* = 0.077).

### Haplotype analysis of the SNPs at rs2270191, rs3132550, rs9264942, and rs6915986

Haplotype frequencies were calculated using an expectation–maximization algorithm for the four SNPs (rs2270191, rs3132550, rs9264942, and rs6915986) and compared among UC, CD, PBC, and healthy control groups. The four-SNP haplotype of TACC, which was predicted as associated with the particular HLA haplotype of HLA-C*12:02~B*52:01~DRB1*15:02, showed a strong susceptibility correlation between UC and controls (OR 2.53; *pc* = 3.96 × 10^–7^), but a protective association between CD and controls (OR 0.50; *pc* = 0.002) (Table 4). The four-SNP haplotype of TATC could not be calculated, which indicated that all subjects carrying the particular HLA haplotype of HLA-C*12:02~B*52:01~DRB1*15:02 had the TACC haplotype, i.e., the SNP at rs9264942 of subjects carrying the HLA haplotype of HLA-C*12:02~B*52:01~DRB1*15:02 was the C allele (CC or CT genotype). The four-SNP haplotype of CGTT in individuals with the SNP T allele at rs9264942 showed a protective correlation between UC and controls (OR 0.56; *pc* = 2.66 × 10^–5^), whereas the four-SNP haplotype of CGCT in individuals with the SNP C allele at rs9264942 displayed no significance (*pc* = 0.803). On the other hand, the four-SNP haplotype of CGTT showed no significance between CD and controls (*pc* = 0.828), while the four-SNP haplotype of CGCT exhibited significant susceptibility (OR 1.31; *pc* = 0.042). The above four-SNP haplotype frequencies were comparable between healthy and PBC disease controls (Table 4).

### Comparisons of clinical findings of the 4-SNP haplotype for IBD

There were no significant differences for any clinical findings between UC patients with and without the TACC haplotype (Supplementary Table 2). In contrast, significant differences were observed for male frequency (OR 2.30, 95% CI 1.06–5.01; *p* = 0.032), disease phenotype location of ileocolonic pattern (OR 3.17, 95% CI 1.44–6.96; *p* = 0.003), intestinal complication frequency (OR 3.40, 95% CI 1.55–7.45; *p* = 0.001), and frequency of history of intestinal surgery (OR 3.33, 95% CI 1.50–7.39; *p* = 0.002) between CD patients with and without the TACC haplotype (Table 5).

**Table 5:** Relationship between haplotypes from four SNPs (rs2270171, rs3132550, rs9264942, and rs6915986) and clinical findings in CD.

	TACC-negative (n = 246)	TACC-positive (n = 29)	*p* value	OR (95% CI)
Male, (%)	175 (71.1%)	15 (51.7%)	0.032	2.30 (1.06–5.01)
Age, years	41 (13–77)	40 (22–87)	0.116	–
Disease duration	15 (1–51)	12 (3–34)	0.116	–
Disease phenotype location: colonic, ileal, ileocolonic, and not determined	36 (14.6%), 40 (16.3%), 170 (69.1%), 0 (0%)	9 (31%), 8 (27.2%), 12 (41.4%), 0 (0%)	0.003	–
Ileocolonic	170 (69.1%)	12 (41.4%)	0.003	3.17 (1.44–6.96)
Age of onset	23 (7–62)	23 (15–64)	0.412	–
Family history of IBD	23 (9.3%)	3 (10.3%)	0.862	0.89 (0.25–3.18)
Medical therapy				
5-ASA	244 (99.2%)	29 (100%)	0.626	–
PSL	97 (39.4%)	11 (37.9%)	0.876	1.07 (0.48–2.35)
Anti-TNF-α administration	134 (54.5%)	16 (55.2%)	0.943	0.97 (0.45–2.11)
Anti-IL-12/23p40	7 (2.8%)	2 (6.9%)	0.246	0.40 (0.08–2.0)
AZA or 6-MP	124 (50.4%)	18 (62.1%)	0.235	0.62 (0.28–1.37)
Anal surgery history	116 (47.2%)	11 (37.9%)	0.346	1.46 (0.66–3.22)
History of intestinal surgery	165 (67.1%)	11 (37.9%)	0.002	3.33 (1.50–7.39)
Balloon expansion	22 (8.9%)	1 (3.4%)	0.312	2.75 (0.36–21.2)
Disease complication				
Presence of anal lesion	161 (65.4%)	16 (55.2%)	0.275	1.54 (0.71–3.35)
Intestinal complication	187 (76.0%)	14 (48.3%)	0.001	3.40 (1.55–7.45)
Extra-intestinal complication	46 (18.7%)	4 (13.8%)	0.517	1.44 (0.48–4.33)
Intestinal carcinoma	2 (0.8%)	0 (0%)	0.626	–

Data are expressed as the number (%) except for age, which is expressed as the median (first-third quartile).

*SNP* single-nucleotide polymorphism, *CD* Crohn’s disease, *OR* odds ratio, *CI* confidence interval, *IBD* inflammatory bowel disease, *5-ASA* 5-aminosalicylic acid, *PSL* prednisolone, *CAP* cytapheresis, *AZA* azathioprine, *6-MP* 6-mercaptopurine, *Tac* tacrolimus, *CyA* cyclosporin.

## Discussion

This study clearly demonstrated a significant association of an eQTL SNP of HLA-C with the regulation of HLA-C expression on PBMC in Japanese subjects. Moreover, four SNP haplotypes consisting of the eQTL SNP of HLA-C and three SNPs of HLA-C*12:02~B*52:01~DRB1*15:02 were significantly related to susceptibility to UC but protection against CD.

The cell surface expression of HLA-C is regulated through multiple mechanisms^30^. Located 35 kb upstream of the *HLA-C* gene (Supplementary Fig. 1), the SNP variant at rs9264942 has been associated with HLA-C surface expression in subjects of European-descent^24,31,32^, but not in an African American population^21^, suggesting that the function of the eQTL SNP at rs9264942 in HLA-C expression varies among population groups. Therefore, we examined for and identified a significant association of the eQTL SNP at rs9264942 with HLA-C expression on PBMC, specifically on CD3e^+^CD8a^+^ lymphocytes, macrophages, and neutrophils, in the Japanese. HLA-C cell surface expression differed among cell populations, which supported previous findings of relatively low HLA-C expression on lymphocytes^33–35^ and high expression on antigen-presenting cells, such as macrophages^36^. The increased expression of HLA-C molecules promotes antigen presentation and recognition by CD8a^+^ cytotoxic T cells and natural killer cells for host protection, but in some instances leads to multiple diseases. HLA-C surface expression is also regulated by microRNA miR-148a binding on the 3′ untranslated region (3′UTR) of the HLA-C gene^37,38^. Therefore, interactions between the eQTL SNP of HLA-C at rs9264942 and miR-148a binding on the 3′UTR region of the *HLA-C* gene merit future consideration to uncover the precise genetic and molecular mechanisms of HLA-C expression. We also compared the HLA-C expression on PBMC between the AA or AG genotype and the GG genotype at rs2395471, which was reported as another eQTL SNP of HLA-C^23^. HLA-C expression on CD3e^+^CD8a^+^ T lymphocytes was significantly higher for the AA or AG genotype than for the GG genotype at rs2395471, but no differences were observed on macrophages and neutrophils. Additional molecular mechanisms of HLA-C expression regulation on PBMC subsets by eQTL SNP differences require assessment in future studies.

Okada et al. reported that the HLA-C*12:02~B*52:01~DRB1*15:02 haplotype was associated with susceptibility to UC as well as protection against CD in the Japanese^25^. If patients had the particular three-SNP haplotype of TAC at rs2270191, rs3132550, and rs6915986, this haplotype showed very similar sensitivity to UC as did the HLA-C*12:02~B*52:01~DRB1*15:02 haplotype^25^. Moreover, the TAC haplotype frequency was low in CD patients, which supported a similar findings for the HLA-C*12:02~B*52:01~DRB1*15:02 haplotype frequency in a previous report^25^. Our result indicated that three-SNP haplotype assignments could serve as surrogate markers for disease susceptibility or protectivity to IBD instead of HLA ones. Although the HLA-C eQTL SNP at rs9264942 was located on this haplotype, the r^2^ values for each SNP were relatively high apart from those between the SNP at rs9264942 and the others of less than 30. This indicated that LD was broken only by rs9264942 and therefore the possibility that the eQTL SNP at rs9264942 had an independent function in terms of IBD susceptibility. Thus, we analyzed the relationship of four-SNP haplotypes with IBD risk. The SNP at rs9264942 was related to CD susceptibility in European patients by the regulation of HLA-C expression^21,24^. We detected an association of the TACC haplotype at rs9264942, but not the TATC haplotype, which suggested that IBD susceptibility might not be related to the eQTL SNP at rs9264942 if patients carried the three SNPs of the TAC haplotype, thereby depending on a particular HLA-C*12:02~B*52:01~DRB1*15:02 haplotype^25^. Reciprocally, the CGTT haplotype in patients with the T allele at rs9264942 (low HLA-C expression group) was associated with UC protection, whereas the CGCT haplotype in patients with the C allele at rs9264942 (high HLA-C expression group) exhibited no remarkable association (Table 4). On the other hand, the CGTT haplotype was not associated with CD, although the CGCT haplotype had a significant association with CD susceptibility. This supported a previous paper describing that high HLA-C expression was related to CD susceptibility^21^. Attempts to confirm the independent involvement of the SNP at rs9264942 in IBD susceptibility or protection by stratified analysis using the Mantel–Haenszel test were inconclusive (data not shown), which might have been due to a relatively small number of subjects or a stronger association of a particular haplotype. Larger studies are needed to validate our findings.

Regarding disease phenotype, it was interesting that CD patients without the TACC haplotype had significantly more intestinal complications (intestinal stenosis, perforation, and fistula) and history of intestinal surgery, suggesting that the HLA-C*12:02~B*52:01~DRB1*15:02 haplotype might impart a stronger effect on clinical results. Based on the above findings, these four SNPs may be simple but useful surrogate markers for predicting HLA-C*12:02~B*52:01~DRB1*15:02 haplotypes as well as disease outcome. Other HLA class II alleles have been associated with IBD onset among populations, such as HLA-DRB1*01:03 with disease predisposition to CD and UC in Europeans, but not in East-Asian Backgrounds^39,40^. Further studies are required to identify the direct mechanisms of involvement of these genetic variants on disease susceptibility and outcome.

The present study had several limitations. First, it evaluated HLA-C expression on the PBMC of healthy controls. As the number of normal controls was too small for a definitive conclusion, a larger validation analysis with more subjects is needed to confirm our results. However, earlier studies have used as few as 30 healthy controls^31^. HLA-C expression in tissue samples should also be analyzed to confirm the molecular mechanisms of HLA-C involvement in IBD. Second, this investigation was preliminary in nature because the numbers of cases and controls were limited. However, we adopted a PBC disease control cohort for the association analysis, whereby the IBD cohort was significantly different from healthy controls as well as from PBC patient controls regarding genetic frequency. Additional investigation is required to validate these newly discovered associations in a larger number of individuals; however, power calculations based on the study subjects of 160 UC patients and 275 CD patients and an effect size of 0.28 at rs9264942 showed sufficient detection power (0.99) at the 0.05 level of significance^41^. Moreover, the calculated type I error and type II were 0.001 and 0.004, respectively, which suggested that statistical error could be avoided. Third, the HLA-C*12:02~B*52:01~DRB1*15:02 haplotype should be examined in more detail to strengthen the results of this study. Fourth, this investigation was retrospective in nature and did not include a segregation analysis; prospective longitudinal studies are needed to clarify the associations of genetic polymorphisms with IBD outcomes.

In conclusion, our findings supported that the eQTL SNP at rs9264942 regulated HLA-C expression in the Japanese. Our association study also implicated four SNPs in strong LD of a particular HLA haplotype, HLA-C*12:02~B*52:01~DRB1*15:02, with IBD susceptibility and outcome, which might be potential surrogate markers for the disease.

## Patients and methods

### Research ethics considerations

This study was conducted in accordance with the principles of the 1975 Declaration of Helsinki and approved by the ethics committees of each participating institution [Shinshu University School of Medicine, Matsumoto, Japan (approval numbers: 639 and 4533), Japanese Red Cross Society Suwa Red Cross Hospital, Suwa, Japan (approval number: 30-18), and Tokyo Yamate Medical Center, Tokyo, Japan (approval number: 188)]. Informed written consent was obtained from all patients and healthy subjects.

### Study subjects

A total of 32 healthy control subjects were included for the analysis of HLA-C expression on PBMC (Table 1). The controls were volunteers from hospital staff who had indicated the absence of any major illnesses and no direct familial relations in a standard questionnaire.

A total of 160 patients with UC and 275 patients with CD who were seen between April 2014 and May 2019 at Shinshu University School of Medicine, Matsumoto, Japan, Japanese Red Cross Society Suwa Red Cross Hospital, Suwa, Japan, or Tokyo Yamate Medical Center, Tokyo, Japan, along with 325 healthy subjects including the above-described 32 healthy control subjects were enrolled for this association study (Table 2). A total of 328 PBC patients who were diagnosed as having PBC at Shinshu University Hospital between 1986 and 2015 were also included as a disease control group in this analysis. No participants were direct relatives of each other, and all were of an East-Asian genetic background.

The diagnoses of UC and CD were based on established Japanese Society of Gastroenterology guidelines using endoscopic, histologic, radiographic, and serologic findings^42^. We also sub-classified the IBD patients into disease parameters based on the phenotype of disease location (colonic, ileal, ileocolonic, and not determined in CD and proctitis, left-sided colitis, pancolitis, and not determined in UC) (Table 2)^2,43,44^. The introduction of prednisolone (PSL) and anti-TNF-α was recommended by the guidelines for moderate or severe UC or CD^42^. Oral or intravenous PSL was advised as a remission induction therapy for left-sided colitis or pancolitis in UC, while anti-TNF-α was advocated for induction or maintenance therapy. A PSL-positive status was designated for patients with a history of PSL administration. A PSL- and anti-TNF-α-positive status was designated for patients who had received both PSL and anti-TNF-α.

The diagnosis of PBC was based on the criteria from the Japan Society of Hepatology^45^. This sub-cohort has been well characterized in prior genetic and clinical studies^46–54^.

### Flow cytometry analysis of HLA-C expression on CD3e^+^CD8a^+^ lymphocytes

PBMC were obtained one day after the hemolysis of red blood cells in ACK lysing buffer (Thermo Fisher Scientific K.K., Tokyo, Japan) according to the manufacturer’s instructions. PBMC were incubated with antibodies that included 7-AAD (BioLegend, San Diego, USA), APC-anti-CD3e (BioLegend), FITC-anti-CD8a (BioLegend), and PE-anti-HLA-C (BD Biosciences) or PE-HLA-C-isotype control by IgG1 k (BioLegend) for 30 min on ice, washed twice with staining buffer, and then analyzed using a BD FACSCanto™ II flow cytometer (BD Biosciences, New Jersey, USA). HLA-C expression was determined by median fluorescence intensity on CD3e^+^CD8a^+^ lymphocytes, CD3e^+^CD8a^−^ lymphocytes, neutrophils, and macrophages in relation to the isotype control. The gating strategy is shown in Fig. 1a. Obtained data were analyzed by FlowJo software (BD Biosciences). All samples were simultaneously stained and analyzed in a one-day experiment.

### SNP determination

Genomic DNA from all participants was isolated from whole blood samples using Quick Gene-610L assays (Fujifilm, Tokyo, Japan). The DNA concentration was adjusted to 10–15 ng/μL for SNP genotyping experiments. Two eQTL SNPs at rs9264942 and rs2395471 were selected based on a previous study^22,23^. Three SNPs at rs2270191, rs3132550, and rs6915986, which were in strong LD with the HLA-C*12:02~B*52:01~DRB1*15:02 haplotype, were also adopted based on an earlier report^22^. The genotyping of five SNPs located at rs9264942, rs2395471, rs2270191, rs3132550, and rs6915986 was performed with a TaqMan 5 exonuclease assay using primers supplied by Applied Biosystems (Foster City, CA, USA). The probe’s fluorescence signal was detected with a StepOne Plus Real-Time PCR System (Thermo Fisher Scientific) according to the manufacturer’s instructions.

### SNP haplotype analysis

Haploview version 4.2^55,56^ was used to evaluate the haplotype structure of the four SNPs at rs2270191, rs3132550, rs9264942, and rs6915986 (Fig. 2). Pairwise LD patterns and haplotype frequency analysis for all SNPs in patients and controls were analyzed by the block definition established by Gabriel et al.^57^.

### Statistical analysis

The significance of associations was evaluated using chi-squared analysis or Fisher’s exact test. *p *values were subjected to Bonferroni’s correction by multiplication by the number of different alleles observed in each locus (*pc*). The Mann–Whitney U-test was used to analyze continuous variables. Stepwise logistic regression analysis with a forward approach was performed to identify independent SNPs associated with IBD susceptibility. A two-sided *p *value of < 0.05 was considered statistically significant. Association strength was estimated by calculating the OR and 95% CI with StatFlex software (version 7.0.3, Artech, Osaka, Japan) as well as IBM SPSS Statistics version 23.0 (IBM, Chicago, IL, USA).

## Supplementary information

Supplementary information.
