# Supplementary material for: Polymorphism at rs9264942 is associated with HLA-C expression and inflammatory bowel disease in the Japanese
Source: Sci Rep. 2020 Jul 24;10:12424. doi: 10.1038/s41598-020-69370-8 (PMC7381613; doi:10.1038/s41598-020-69370-8)

Polymorphism at rs9264942 is associated with HLA-C expression and inflammatory bowel disease in the Japanese

Hiroshi Suzuki, Satoru Joshita, Atsuhiro Hirayama, Akihiro Shinji, Kenji Mukawa, Minako Sako, Naoki Yoshimura, Tomoaki Suga, Takeji Umemura, Norihiro Ashihara, Tomoo Yamazaki, and Masao Ota

Table of contents

Supplementary Table 1……………………………………………………………2-3

Supplementary Table 2……………………………………………………………4-5

Supplementary Table 3……………………………………………………………6-7

Supplementary Figure 1…………………………………………………………… 8

**Supplementary Table 1.** **Relationship between HLA-C expression and clinical findings in UC**

|  | TT  (n=37) | CC/CT  (n=123) | *p-*value | OR (95%CI) |
| --- | --- | --- | --- | --- |
| Male, (%) | 20 (54.1%) | 63 (51.2%) | 0.762 | 1.12 (0.54-2.34) |
| Age, years | 51 (27-84) | 47 (19-80) | 0.218 | - |
| Disease duration | 11.5 (1-36) | 11 (1-43) | 0.890 | - |
| Disease phenotype location: proctitis, left-sided colitis, pancolitis, not determined | 5 (13.5%), 19 (51.4%), 13 (35.1%), 0 (0%) | 26 (21.1%), 36 (29.3%), 60 (48.8%), 1 (0.8%) | 0.48 | - |
|  |  |  |  |  |
| Pancolitis | 13 (35.1%) | 60 (48.8%) | 0.144 | 0.57 (0.27-1.22) |
| Age of onset | 38 (20-68) | 34 (9-76) | 0.109 | - |
| Family history of IBD | 1 (2.7%) | 4 (3.3%) | 0.866 | 0.83 (0.09-7.63) |
| Medical therapy |  |  |  |  |
| 5-ASA internal use | 37 (100%) | 122 (99.2%) | 0.582 | - |
| 5-ASA enema | 2 (5.6%) | 7 (5.7%) | 0.975 | 0.98 (0.19-4.91) |
| PSL oral | 20 (55.6%) | 64 (52.5%) | 0.744 | 1.13 (0.54-2.39) |
| PSL enema | 21 (58.3%) | 58 (47.5%) | 0.255 | 1.55 (0.73-3.28) |
| PSL resistance | 2 (5.6%) | 13 (10.6%) | 0.365 | 0.50 (0.11-2.32) |
| Anti-TNF-α administration | 11 (30.6%) | 37 (30.3%) | 0.979 | 1.01 (0.45-2.27) |
| Anti-TNF-α+PSL | 10 (27.8%) | 32 (26.2%) | 0.853 | 1.08 (0.47-2.49) |
| CAP | 5 (13.9%) | 23 (18.9%) | 0.493 | 0.69 (0.24-1.98) |
| AZA or 6-MP | 13 (36.1%) | 47 (38.5%) | 0.793 | 0.90 (0.42-1.95) |
| Tac / CyA | 3 (8.3%) | 11 (9%) | 0.899 | 0.92 (0.24-3.48) |
| History of intestinal surgery | 2 (5.4%) | 10 (8.1%) | 0.581 | 0.64 (0.14-3.09) |
| Disease complication |  |  |  |  |
| Intestinal complication | 0 (0%) | 3 (2.4%) | 0.338 | - |
| Extra-intestinal complications | 3 (8.1%) | 3 (2.4%) | 0.112 | 3.53 (0.68-18.3) |
| Intestinal carcinoma | 0 (0%) | 1 (0.8%) | 0.582 | - |

Abbreviations: HLA, human leukocyte antigen; UC, ulcerative colitis; OR, odds ratio; CI, confidence interval; IBD, inflammatory bowel disease; 5-ASA, 5-aminosalicylic acid; PSL, prednisolone; CAP, cytapheresis; AZA, azathioprine; 6-MP, 6-mercaptopurine; Tac, tacrolimus; CyA, cyclosporin. Data are expressed as the number (%) except for age, which is expressed as the median (first-third quartile).

**Supplementary Table 2.** **Relationship between haplotypes from four SNPs (rs2270171, rs3132550, rs9264942, and rs6915986) and clinical findings in UC**

|  | TACC-negative  (n=89) | TACC-positive  (n=71) | *p-*value | OR (95%CI) |
| --- | --- | --- | --- | --- |
| Male, (%) | 51 (57.3%) | 32(45.1%) | 0.124 | 1.64 (0.87-3.07) |
| Age, years | 50 (19-84) | 47(19-80) | 0.438 | - |
| Disease duration | 10.5 (1-41) | 11 (1-43) | 0.823 | - |
| Disease phenotype location: proctitis, left-sided colitis, pancolitis, not determined | 15 (16.9%), 33 (37.1%), 41 (46.1%), 0 (0%) | 16 (22.5%), 22 (31.0%), 32 (45.1%), 1 (1.4%) | 0.68 | - |
|  |  |  |  |  |
| Pancolitis | 41 (46.1%) | 32 (45.1%) | 0.90 | 1.04 (0.56-1.95) |
| Age of onset | 36 (14-68) | 32 (9-76) | 0.253 | - |
| Family history of IBD | 2 (2.2%) | 3 (4.2%) | 0.475 | 0.52 (0.09-3.21) |
| Medical therapy |  |  |  |  |
| 5-ASA internal use | 89 (100%) | 70 (98.6%) | 0.261 | - |
| 5-ASA enema | 3 (3.4%) | 6 (8.5%) | 0.171 | 0.38 (0.09-1.59) |
| PSL oral | 47 (53.4%) | 37 (52.9%) | 0.945 | 1.02 (0.55-1.92) |
| PSL enema | 46 (52.3%) | 33 (47.1%) | 0.522 | 1.23 (0.66-2.30) |
| PSL resistance | 8 (9.1%) | 7 (9.9%) | 0.869 | 0.91 (0.32-2.66) |
| Anti-TNF-α　administration | 25 (28.4%) | 23 (32.9%) | 0.546 | 0.81(0.41-1.60) |
| Anti-TNF-α+PSL | 23 (26.1%) | 19 (27.1%) | 0.887 | 0.95 (0.47-1.93) |
| CAP | 14 (15.9%) | 14 (20%) | 0.504 | 0.76 (0.33-1.72) |
| AZA or 6-MP | 31 (35.2%) | 29 (41.4%) | 0.425 | 0.77 (0.40-1.47) |
| Tac / CyA | 7 (8%) | 7 (10%) | 0.653 | 0.78 (0.26-2.33) |
| History of intestinal surgery | 5 (5.6%) | 7 (9.9%) | 0.312 | 0.54 (0.17-1.79) |
| Disease complication |  |  |  |  |
| Intestinal complication | 2 (2.2%) | 1 (1.4%) | 0.698 | 1.61 (0.14-18.1) |
| Extra-intestinal complication | 3 (3.4%) | 3 (4.2%) | 0.777 | 0.79 (0.16-4.04) |
| Intestinal carcinoma | 0 (0%) | 1 (1.4%) | 0.261 | - |

Abbreviations: SNP, single-nucleotide polymorphism; UC, ulcerative colitis; OR, odds ratio; CI, confidence interval; IBD, inflammatory bowel disease; 5-ASA, 5-aminosalicylic acid; PSL, prednisolone; CAP, cytapheresis; AZA, azathioprine; 6-MP, 6-mercaptopurine; Tac, tacrolimus; CyA, cyclosporin. Data are expressed as the number (%) except for age, which is expressed as the median (first-third quartile).

**Supplementary Table 3.** **Relationship between HLA-C expression and clinical findings in CD**

|  | TT  (n=107) | CT/CC  (n=168) | *p-*value | OR (95%CI) |
| --- | --- | --- | --- | --- |
| Male, (%) | 77 (72%) | 113 (67.3%) | 0.411 | 1.25 (0.74-2.12) |
| Age, years | 42 (17-77) | 41 (13-87) | 0.736 | - |
| Disease duration | 15 (1-51) | 14 (1-39) | 0.411 | - |
| Disease phenotype  location: colonic, ileal, ileocolonic, and not determined | 13 (12.1%), 17 (15.9%), 77 (72%), 0 (0%) | 32 (19%), 31 (18.5%), 105 (62.5%), 0 (0%) | 0.22 | - |
|  |  |  |  |  |
| Ileocolonic | 77 (72%) | 105 (62.5%) | 0.105 | 1.54 (0.91-2.60) |
| Age of onset | 22 (8-48) | 24 (7-64) | 0.228 | - |
| Family history of IBD | 10 (9.3%) | 16 (9.5%) | 0.961 | 0.98 (0.43-2.25) |
| Medical therapy |  |  |  |  |
| 5-ASA | 107 (100%) | 166 (98.8%) | 0.257 | - |
| PSL | 42 (39.3%) | 66 (39.3%) | 0.996 | 1.00 (0.61-1.64) |
| Anti-TNF-α　administration | 60 (56.1%) | 90 (53.6%) | 0.684 | 1.11 (0.68-1.80) |
| Anti-IL-12/23p40 | 6 (5.6%) | 3 (1.8%) | 0.082 | 3.27 (0.80-13.4) |
| AZA or 6-MP | 52 (48.6%) | 90 (53.6%) | 0.421 | 0.82 (0.50-1.33) |
| Anal surgery history | 50 (46.7%) | 77 (45.8%) | 0.885 | 1.04 (0.64-1.69) |
| History of intestinal surgery | 72 (67.3%) | 104 (61.9%) | 0.364 | 1.27 (0.76-2.11) |
| Balloon expansion | 6 (5.6%) | 17 (10.1%) | 0.188 | 0.53 (0.20-1.38) |
| Disease complication |  |  |  |  |
| Presence of anal lesion | 74 (69.2%) | 103 (61.3%) | 0.185 | 1.42 (0.85-2.37) |
| Intestinal complication | 85 (79.4%) | 116 (69%) | 0.058 | 1.73 (0.98-3.07) |
| Extra-intestinal complication | 17 (15.9%) | 33 (19.6%) | 0.431 | 0.77 (0.41-1.47) |
| Intestinal carcinoma | 0 (0%) | 2 (1.2%) | 0.257 | - |

Abbreviations: HLA, human leukocyte antigen; CD, Crohn’s disease; OR, odds ratio; CI, confidence interval; IBD, inflammatory bowel disease; 5-ASA, 5-aminosalicylic acid; PSL, prednisolone; CAP, cytapheresis; AZA, azathioprine; 6-MP, 6-mercaptopurine; Tac, tacrolimus; CyA, cyclosporin. Data are expressed as the number (%) except for age, which is expressed as the median (first-third quartile).

Supplementary Figure 1.

Interrelationship of SNPs at rs9264942, rs2270191, rs3132550, and rs6915986 along with the HLA-C, HLA-B, and HLA-DRB1 gene in the HLA region (6p21.31). The eQTL SNP of rs9264942 is located 35 kb upstream of the coding region of the HLA-C gene. Imputed SNPs at rs2270191, rs3132550, and rs6915986 had strong LD with *HLA-C*12:02* (r^2^ = 1), *HLA-B*52:01* (r^2^ = 0.94), and *HLA-DRB1*15:02* (r^2^ = 0.89) alleles, respectively.


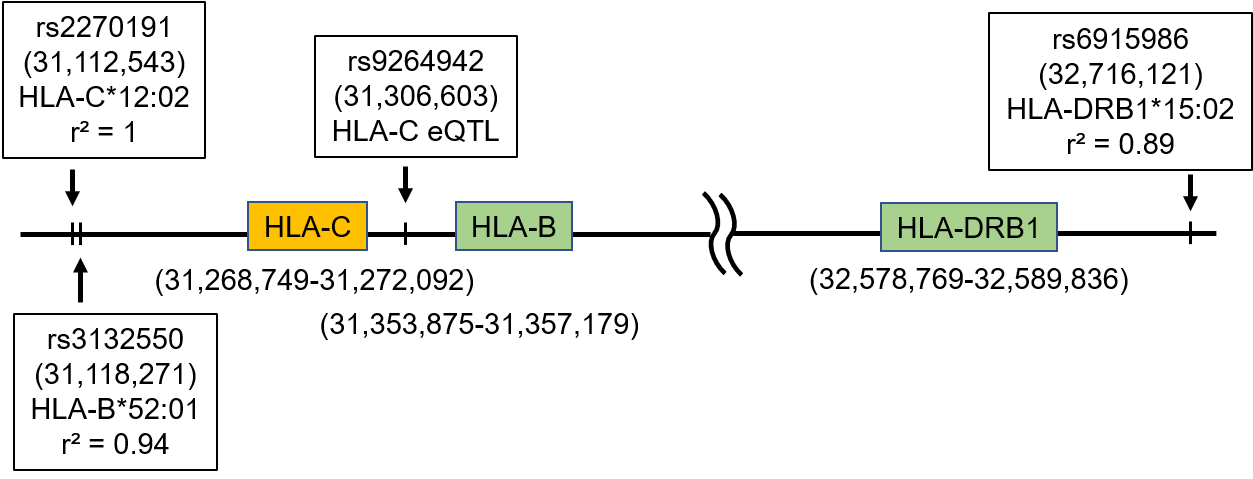

Supplement: Supplementary file 1 — Supplementary information. [file 41598_2020_69370_MOESM1_ESM.docx]
